# *De novo* genome assembly of a probiotic *Lacticaseibacillus rhamnosus* ISO20, isolated from raw milk in South Africa

**DOI:** 10.1128/mra.01227-23

**Published:** 2024-02-20

**Authors:** Goitsemang Makete, Tshifhiwa Paris Mamphogoro

**Affiliations:** 1 Gastro-Intestinal Microbiology and Biotechnology Unit, Agricultural Research Council-Animal Production, Irene, Pretoria, South Africa; Wellesley College Department of Biological Sciences, USA

**Keywords:** *Lacticaseibacillus rhamnosus*, goat's milk, potential probiotic bacterium, facultative anaerobic heterofermentative bacteria, *de novo* assembly

## Abstract

Lactic acid bacteria are known to exhibit probiotic properties through various mechanisms including production of antimicrobial substances and bile salts tolerance. Here, we report a draft genome sequence of *Lacticaseibacillus rhamnosus* ISO20, a lactic acid bacterium isolated from raw goat’s milk to provide genomic insight into its strategies as probiotic strain.

## ANNOUNCEMENT

*Lacticaseibacillus rhamnosus* is a facultative anaerobic heterofermentative rod-shaped bacterium isolated from different ecological niches, such as gastrointestinal tract, fermented dairy products, and plant-associated environment (1–3). *L. rhamnosus* has a long safety history of applications where health and industrial benefits are associated with different strains (4).

*L. rhamnosus* ISO20 was isolated from goat’s milk sourced at the small-stock Division of the Agricultural Research Council, Animal Production (Irene, South Africa; 25° 53*'* 59.6*"*S 28° 12*'* 51.6*"*E). Forty raw milk samples were collected and enclosed in sterile plastic containers and transported on ice to the laboratory. One milliliter of each milk sample was suspended in 9 mL of sterile saline solution (0.85% wt/vol NaCl), and the mixture was serially diluted up to 10^−5^. The sample suspension was then inoculated onto De Man, Rogosa, and Sharpe (MRS) agar supplemented with 0.05 g/L cysteine-HCL (MRS-cysHCL) and incubated for 24–48 hours at 37°C under anaerobic conditions. Distinct colonies formed on the plates were selected. Pure strain was obtained by subculturing onto sterile MRS-cysHCL (5).

The genome of ISO20 was extracted from overnight liquid culture using the Quick-DNA Fungal/bacterial Miniprep Kit (Zymo Research, Irvine, CA) following the manufacturer’s instructions. The DNA concentration was measured using a NanoDrop (ThermoFisher Scientific, Carlsbad, CA, USA), and DNA quality was evaluated on 2% agarose gel. The paired-end (2 × 150 bp) libraries were generated using the EBNext Ultra II FS DNA Library Prepkit (New England Biolabs, Ipswich, MA) and sequenced on an Illumina NextSeq platform at Inqaba Biotechnical Industries (Pty) Ltd. (Pretoria, South Africa), yielding a total of 1,519,878 paired-end reads. The reads quality was evaluated using FastQC v0.11.5 (6) via KBase (7); the raw reads were then trimmed to remove low-quality reads and sequence adaptors using Trimmomatic v0.36 (8). The trimmed reads were *de novo* assembled using SPAdes v3.15.3 (9). Assembly quality was assessed using QUAST v5.0.2 (Table 1) (10). While the genome completeness and contamination were evaluated using CheckM v1.0.18 (11).

**TABLE 1:** Quast assembly statistics

Assembly	SPAdes—v3.15.3
No. of contigs	30
Largest contig	831,503
Total length	2,957,989
GC (%)	46.5
*N*_*50*_	251,390
*N*_75_	141,836
*L*_50_	4
*L*_75_	6
# Ns per 100 kbp	13.15

**Fig 1:**
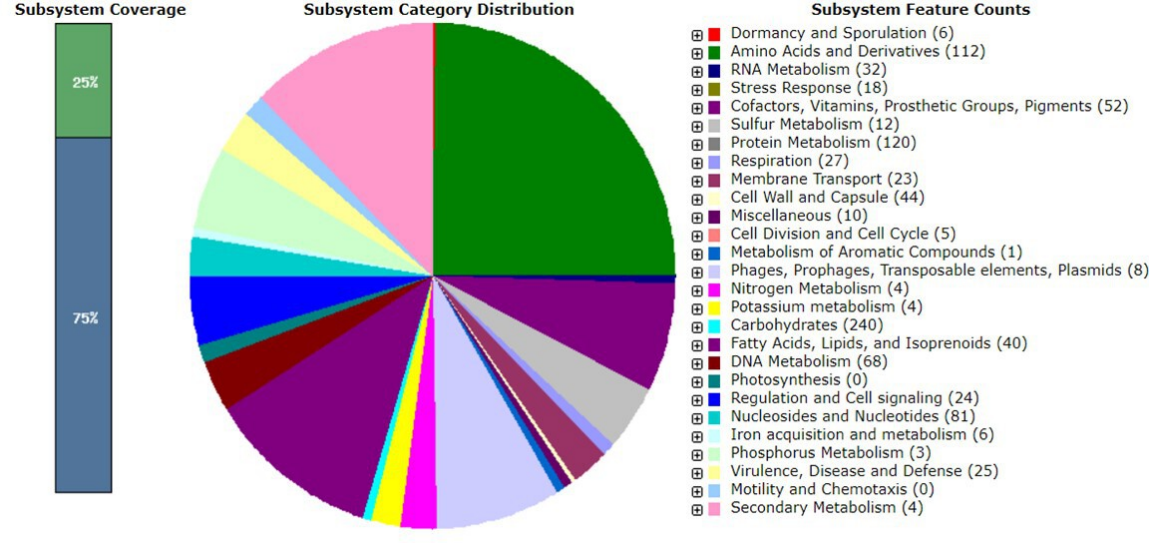
Subsystem category distribution of key PCG of *L. rhamnosus* strain ISO20 annotated in the RAST SEED viewer annotation online server. The green/blue bar represents the subsystem coverage in percentage. Blue bar correlates with the percentage (%) of proteins present.

Identification of ISO20 was conducted using Kaiju v1.7.3 (12), and the results were visualized using Krona v2.7.1 (13). The assembly yielded a genome sequence of 2,957,989 bp long, a G + C content of 46.5%, and a coverage of 154×. Genome completeness was estimated at 98.56%, comprising 30 contigs, with *N*_50_ and *L*_50_ values of 251,390 bp and 4, respectively. Gene annotation was performed using the RASTtk v1.073 and the NCBI Prokaryotic Genome Annotation Pipeline v6.5 (14, 15). All software programs were run with default parameters. Furthermore, genome analysis revealed 2,751 total genes and 62 RNAs. The subsystem statistics showed 27 subsystem feature counts of the coding protein into functional groups with a total of 2,646 Polycomb-group (PCG). The 969 genes were grouped into biological processes, cellular components, and molecular function. The topmost three groups were protein metabolism (*n* = 120), carbohydrates (*n* = 240), and amino acids and derivatives (*n* = 112) (Fig. 1). The RASTtk revealed the presence of genes encoding for acid tolerance, antioxidant, bile salt tolerance, adhesion, and bacteriocin production, all of which are essential characteristics for potential probiotic strains (16).

## Data Availability

This whole-genome shotgun project has been deposited at DDBJ/ENA/GenBank under the accession number JASVVP000000000. The version described in this paper is the first version. The SRA accession number is SRR24904735, the BioProject accession number is PRJNA896361, and the BioSample accession number is SAMN35721675.
